# Nafion®-coated mesoporous Pd film toward remarkably enhanced detection of lactic acid†

**DOI:** 10.1039/c7ra13026k

**Published:** 2018-03-14

**Authors:** Daisuke Baba, Asep Sugih Nugraha, Muhammad Iqbal, Jiang Bo, Cuiling Li, Abdulmohsen Ali Alshehri, Jungmok You, Victor Malgras, Yusuke Yamachi, Toru Asahi

**Affiliations:** International Center for Materials Nanoarchitectonics (WPI-MANA), National Institute for Materials Science (NIMS) 1-1 Namiki Tsukuba Ibaraki 305-0044 Japan MALGRAS.Victor@nims.go.jp tasahi@waseda.jp; Faculty of Science and Engineering, Waseda University 3-4-1 Okubo, Shinjuku Tokyo 169-8555 Japan; Department of Chemistry, King Abdulaziz University P. O. Box. 80203 Jeddah 21589 Saudi Arabia; Department of Plant & Environmental New Resources, Kyung Hee University 1732 Deogyeong-daero, Giheunggu Yongin-si Gyeonggi-do 446-701 South Korea; School of Chemical Engineering, Australian Institute for Bioengineering and Nanotechnology (AIBN), The University of Queensland Brisbane QLD 4072 Australia y.yamauchi@uq.eudu.au

## Abstract

Mesoporous metal films can detect biomarkers with high sensitivity. Further coating the mesoporous metal with polymers enhances sensing selectivity by favoring specific biomarkers against other interferents. In the present study, we report the fabrication of a Nafion®-coated mesoporous Pd film to filtrate interferents present in sweat during non-invasive biosensing. By using a Nafion®-coated mesoporous Pd film, lactic acid, a metabolite present in sweat, can be successfully detected with high sensitivity.

## Abstract 2

Mesoporous metal films can detect biomarkers with high sensitivity.

## Graphical abstract

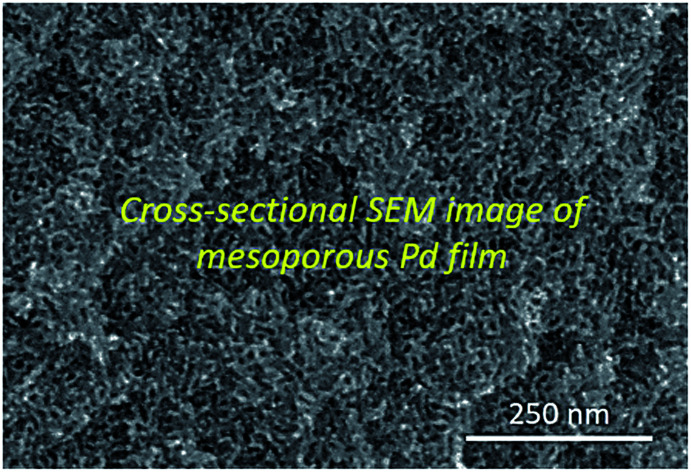

## Introduction

Non-invasive sensing of biomarkers present in sweat, urine and saliva, for early diagnosis of various diseases and health management, is a research topic which has recently attracted considerable attention.^1^ Compared with invasive sensing such as blood collection, non-invasive sensing can reduce damage to and discomfort of the patients, which makes them highly coveted; therefore numerous reports on non-invasive bio-sensing have been released. In particular, sweat can be easily collected from patients and contains various biomarkers which, if detected in time, can contribute to early diagnosis of diseases. For example, tattoo-type, sheet-type and small integrated device-type sensors have previously been reported.^2^

These reported devices use enzymes to detect biomarkers from sweat without having to filtrate any interferents. Despite the presence of interfering species in the medium, enzymes can form enzyme–substrate complexes with specific biomarkers, resulting in high selectivity. However, these sensors have some disadvantages in terms of cost and stability (requiring suitable pH and temperature to prevent degradation), which make their practical use less attractive. On the other hand, non-enzymatic sensors, such as mesoporous metal films and nanoparticles, have recently been reported as a promising alternative.^3^ Compared with enzymatic sensors, their selectivity is still relatively low because they cannot form complexes with biomarkers. Under the application of an optimal voltage, however, the influence of interferents can be reduced. In addition, high sensitivity can be achieved because of the large surface area reacting with the biomarkers.

The typical biomarkers in sweat are lactic acid, glucose, interleukin and other proteins.^4^ Among these biomarkers, lactic acid is one of the metabolites whose concentration in sweat is different before and after exercise.^5^ Furthermore, the relationship between the lactic acid content and the metabolic activity is well-known.^6^ Therefore, if a biosensor could detect and monitor the exact content of lactic acid in sweat, it could be used for health management purposes.

In this study, we aim to detect biomarkers from sweat after filtrating out the interferents with a Nafion®-coated mesoporous Pd film. Nafion® is a polymer which is typically used for ion-exchange.^7^ Mesoporous Pd films can be fabricated in nonionic solution consisting of P123 surfactant and PdCl_2_ metal precursor. The resulting large surface area is suitable for applications in biosensor. Bare mesoporous Pd films have already a strong potential for biosensing, but further coating the film with a Nafion® layer with mesoporous roughness is critical to remove interferents present in sweat.

Although Nafion® has been utilized as a part of electrode materials for biosensing,^8^ the hybrid structure consisting of a mesoporous metal combined with a Nafion® layer has never been reported. Composites made of mesoporous materials and polymers have previously been reported for drug delivery and photochemical applications,^9^ but not for electrochemical biosensing. Integrating filtration and detection in one functional system using mesoporous metal films is a novel approach. Filtration generally needs complicated process, but Nafion® coating is simple and can be used in a “disposable device” technology. In this study, after filtration by Nafion®, lactic acid is monitored by chronoamperometry under voltage which is tuned to minimize the influence of interferents such as glucose, urea, ammonia and ethanol present in sweat. To benchmark the sensitivity of our Nafion®-coated mesoporous Pd film, bare mesoporous Pd, Nafion®-coated nonporous Pd and bare nonporous Pd films are also compared.

## Experimental

### Materials synthesis

The mesoporous Pd film was prepared according to a method we reported previously.^10^ At first, 80 mg of P123 (poly(ethylene oxide)-*block*-poly(propylene oxide)-*block*-poly(ethylene oxide), PEO_20_-PPO_70_-PEO_20_) was dissolved in 2 mL water, followed by the slow addition of 2 mL PdCl_2_ (80 mM) under constant stirring. The solution was then sonicated continuously for 20 min. The three-electrode system used to prepare the mesoporous Pd films consisted of an Ag/AgCl reference electrode, a Pt counter electrode and an Au-coated Si substrate (3 mm × 3 mm) as working electrode. The electrodeposition was performed at an applied potential of 0.0 V for 600 s. After deposition, the Au-coated Si substrate became black. The working electrode was withdrawn quickly from the system and washed with water to remove the remaining P123. The mesoporous Pd film was coated by dropping 10 μL of Nafion® perfluorinated resin solution which was then dried under a lamp. These steps were repeated 12 times.

### Materials characterization

Scanning electron microscope (SEM) imaging of the mesoporous Pd films were obtained using a Hitachi FESEM SU-8000 microscope at an accelerating voltage of 5 kV. Wide-angle X-ray diffraction (XRD) patterns were acquired with a SmartLab Rigaku (Cu Kα radiation; operating voltage 40 kV and current 30 mA). Low-angle XRD patterns were obtained using a Rigaku NANO-Viewer (Cu Kα radiation) equipped with a camera length of 700 mm, an operation voltage of 40 kV and a current of 30 mA. All electrochemical measurements were recorded with a CHI 842B electrochemical analyzer (CHI Instruments, USA).

## Results and discussion

**Fig. 1:**
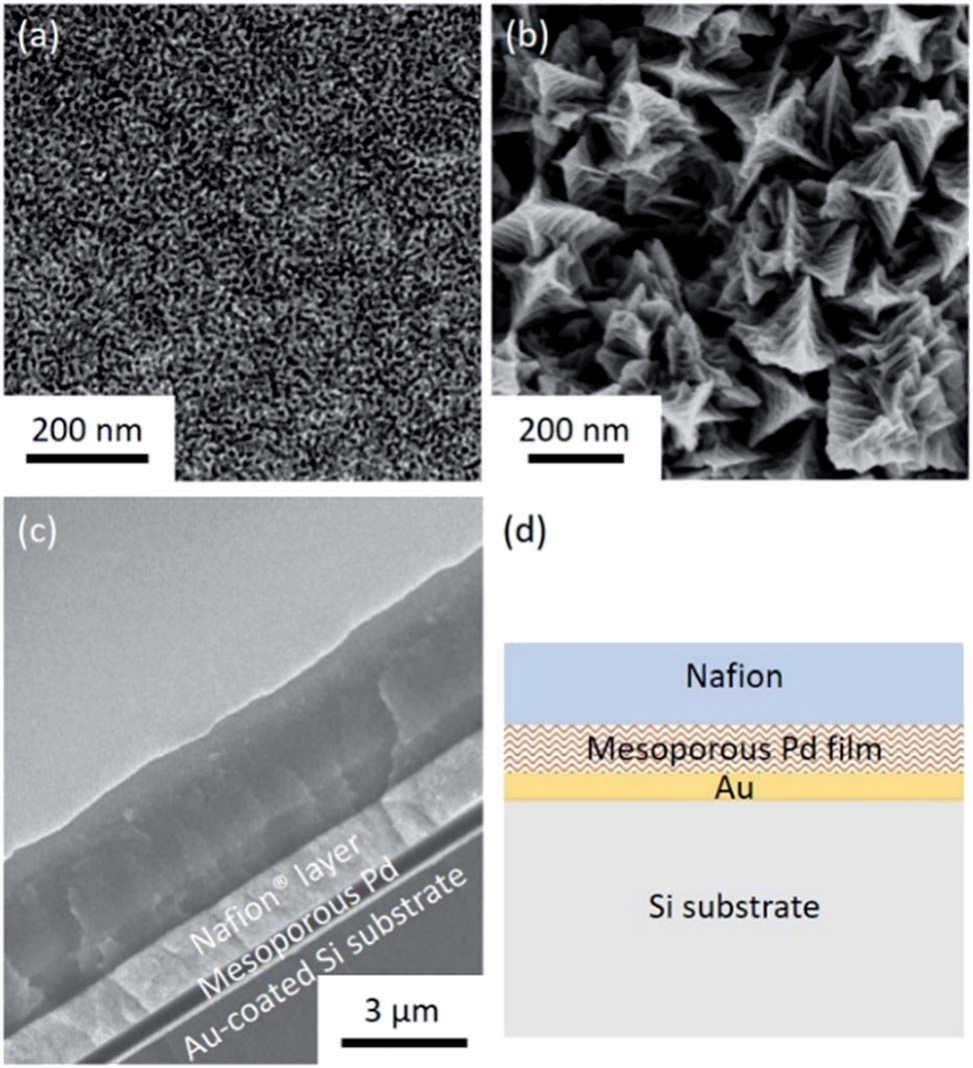
(a) Top-view SEM image of a typical mesoporous Pd film prepared by electrodeposition at 0.0 V *vs.* Ag/AgCl for 600 s. (b) Top-view SEM image of nonporous Pd film prepared by electrodeposition from electrolyte solution in the absence of surfactant at 0.0 V *vs.* Ag/AgCl for 600 s. (c) Cross-sectional SEM image of the Nafion®-coated mesoporous Pd film. (d) Schematic structure of the Nafion®-coated mesoporous Pd film.

Both the top-surface and cross-section of the obtained films are imaged by SEM after carefully removing the surfactants (Fig. 1a and S1†). The SEM images reveal that the pores are uniformly distributed over the entire film. The periodicity of the pore organization can be determined by low-angle XRD. The clear peak centered at 0.62° corresponds to a pore-to-pore distance of *ca.* 14.2 nm (Fig. S2†). From the high resolution SEM image shown in Fig. S3,† the average pore diameter is measured to be *ca.* 10.9 nm (calculated from over 200 pores). The average wall thickness is estimated to be *ca.* 3.3 nm which is consistent with the average wall thickness measured from SEM (Fig. S3†). In the absence of nonionic surfactant, large Pd crystals are formed (Fig. 1b). The cross-sectional SEM image of Nafion®-coated mesoporous Pd film is shown in Fig. 1c. The thickness of the mesoporous Pd film is approximately 600 nm, from which the growth rate is calculated to be about 100 nm min^−1^. Fig. 1d shows the schematic structure of the Nafion®-coated mesoporous Pd film. From Fig. 1c, uniform Nafion® coating was confirmed with a thickness about 1.5 μm. Nafion® is an ion-exchange membrane containing anionic sulphonate groups which can combine with cationic species. The XRD pattern of mesoporous Pd film contains diffraction peaks located at 40.18°, 46.72°, 68.16°, 82.18° and 86.82°, which can be assigned to the (111), (200), (220), (311) and (222) diffraction planes of Pd with a fcc structure, respectively (JCPDS card no. 05-0681, Fig. S4†).

The electrochemical response of the mesoporous Pd films, with and without Nafion® coating were measured in NaCl solution (0.4%) to mimic the typical composition of human sweat. The CV measured in NaCl solution is shown in Fig. S5† and the area under the CV curve for the Nafion®-coated film was smaller than that for the uncoated film. This can be explained as the anionic Nafion® layer has abundant sulphonate groups as well as anionic ions such as chloride, which cannot easily penetrate the membrane. For the bare mesoporous Pd film, the peak observed at around 0.25 V is due to the oxidation of Pd. For reference, Pd in KCl solution is oxidized into K_2_PdCl_4_ with an oxidation peak observed at around 0.25 V.^11^ Furthermore, the smooth peaks spanning from −0.2 V to −0.6 V are also reported in the reference and can be ascribed the reduction of Pd salt into Pd.^11^ These peaks cannot be observed when the mesoporous film is coated with Nafion® which prevents Pd from redox reaction. Previous works reported that protein adhesion is hindered by the presence of Nafion® coating.^12^ Only small molecules such as lactate and glucose can be transported through this polymer,^13^ which is why good selectivity is expected for lactic acid sensing.

**Fig. 2:**
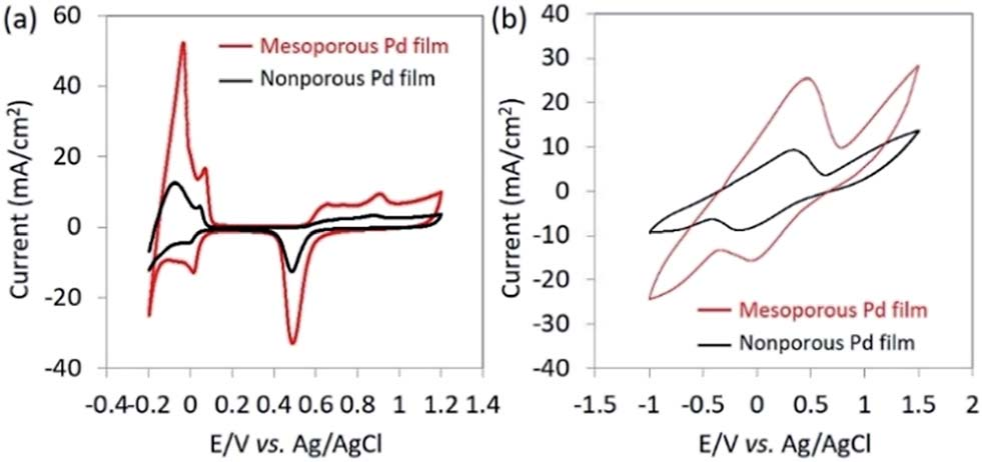
(a) CV curves of bare mesoporous Pd film and bare nonporous Pd films measured in 0.5 M H_2_SO_4_ solution at a scan rate of 50 mV s^−1^. (b) CV curves of bare mesoporous Pd film and bare nonporous Pd films measured in 0.4% NaCl solution containing lactic acid (10 mM). The currents are normalized by the geometric electrode area.

The electrochemical surface area (ECSA) of the catalyst films were investigated by carrying out CV in 0.5 M H_2_SO_4_ with a scan rate of 50 mV s^−1^ between −0.2 and 1.2 V (*vs.* Ag/AgCl) (Fig. 2a). The ECSA is estimated by calculating the charge associated with PdO reduction between the potential of 0.35 and 0.70 V (*vs.* Ag/AgCl) from the negative potential scan of the CV. By assuming that the conversion factor for an oxide monolayer reduction is 420 μC cm^−2^ on a smooth Pd surface, the mass-normalized ECSA for mesoporous Pd films is calculated to be 37.9 m^2^ g^−1^, which is 10 times higher than that of the nonporous Pd films (3.56 m^2^ g^−1^). As shown in Fig. 2b, CVs are measured in NaCl solution containing lactic acid (10 mM) by using mesoporous and nonporous Pd films as the working electrode. Anodic current and cathodic peaks can be observed, corresponding respectively to the oxidation of lactic acid and reduction of pyruvic acid. The electro-oxidation of lactic acid into pyruvic acid is likely to occur at the surface of the Pt electrode, as previously reported.^14^ Because of a high ECSA, the mesoporous film displays larger current peaks and superior catalytic activity towards the oxido-reduction reaction of lactic acid and pyruvic acid compared to that of the nonporous film. Both the dependence of the ECSA and anodic peak current density by lactic acid oxidation on the deposition time are shown in Fig. S6.† Since the size of lactic acid molecules is much bigger than that of H^+^ ion, the anodic peak current density due to oxidation of lactic acid becomes saturated when the film thickness is larger than 600 nm. Therefore, we used the mesoporous Pd film prepared for 600 s for further electrochemical study.

**Fig. 3:**
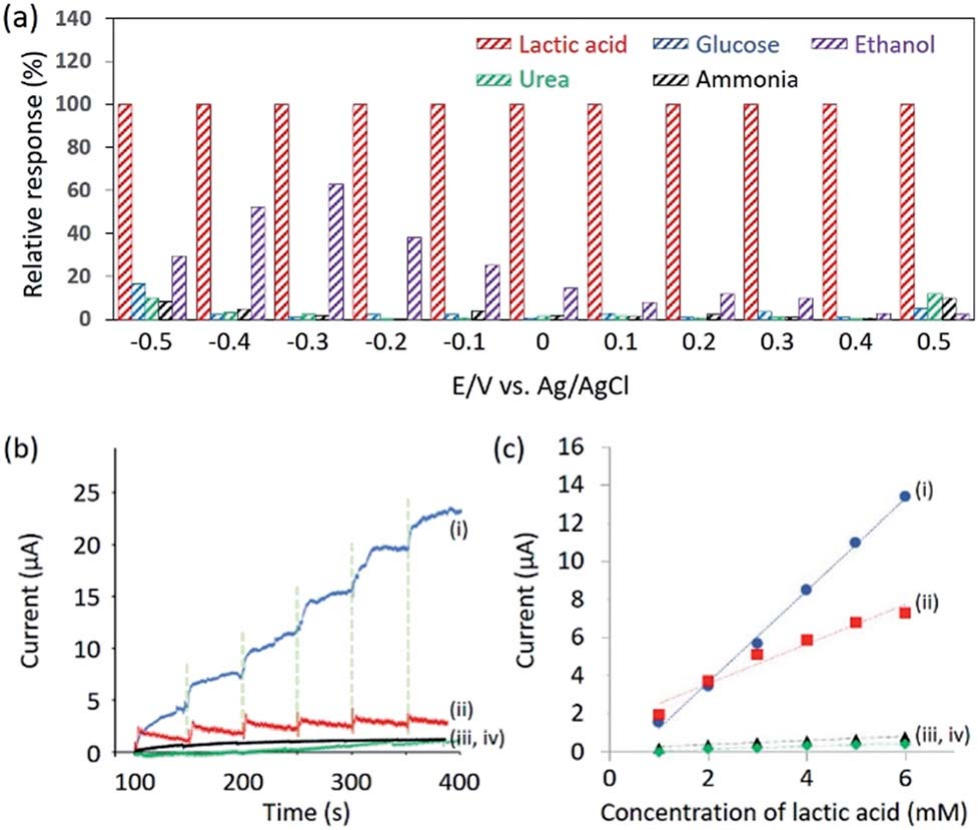
(a) Electrochemical relative response to 0.4% NaCl solution containing lactic acid (1 mM), glucose (0.1 mM), urea (0.1 mM), ammonia (0.1 mM), and ethanol (0.1 mM) solutions, respectively. The electrolyte is 0.4% NaCl solution. (b) Dynamic response of the sensors based on (i) Nafion®-coated mesoporous Pd film, (ii) bare mesoporous Pd film, (iii) Nafion®-coated nonporous Pd film, and (iv) bare nonporous Pd film upon successive addition of 0.4% NaCl solution containing lactic acid (1 mM). The electrolyte is 0.4% NaCl solution. (c) Relationship between the amperometric response and lactic acid concentrations (up to 6 mM).

From Fig. 2b, it can be observed that the oxidation of lactic acid and reduction of pyruvic acid on mesoporous Pd film take place between −0.5 V and 0.5 V (*vs.* Ag/AgCl). Therefore, the chronoamperometry is measured under the same voltage window to minimize the influence of glucose, urea, ammonia or ethanol. These interferents, present in human sweat,^15^ and cannot be removed by the Nafion® coating because they are too small to be filtrated. The current response due to lactic acid and other interferents are compared in Fig. 3a, thus confirming that the contribution from the interferents becomes negligible under an applied potential of 0.4 V (*vs.* Ag/AgCl).

Therefore, chronoamperometry was measured at each addition of 1 mM of lactic acid and the results are shown in Fig. 3b. To evaluate the sensitivity of the Nafion®-coated mesoporous Pd film to lactic acid, bare mesoporous Pd, Nafion®-coated nonporous Pd and bare nonporous Pd films were also tested. The limit of detection (LOD) is calculated to be 0.34 mM (for the Nafion®-coated mesoporous Pd film), 0.48 mM (for the bare mesoporous Pd film), 0.75 mM (for the Nafion®-coated nonporous Pd film), and 1.5 mM (for the bare nonporous Pd film). The LOD using Nafion®-coated mesoporous Pd film (0.34 mM) is superior to what was previously reported (0.5 mM).^16^ The relationship between the concentration of lactic acid and the current is shown in Fig. 3c. The mesoporous Pd film shows a higher sensitivity than the nonporous Pd film, due to a higher ECSA. Interestingly, the Nafion®-coated mesoporous Pd film shows a higher sensitivity than the bare mesoporous Pd film. To discuss this result, adsorption of lactic acid on both electrodes in NaCl solution should be considered. In 0.4% NaCl solution, the pH is almost 7.0 and lactic acid releases H^+^ ion because the p*K*_a_ is 3.86. Therefore, negatively charged lactic acid ions cannot approach the Nafion® surface due to electrostatic repulsion. It would then be expected that the bare mesoporous Pd film shows higher sensitivity. This is due to chloride ions being critical interferents during the sensing of lactic acid. When Nafion® is present, the chloride ions are removed. We tested the repeatability of this sensor using the relationship between the amperometric response and the lactic acid concentration. Even after addition of 15 mM, the linearity is maintained showcasing high repeatability (Fig. S7†).

## Conclusion

We successfully prepared a Nafion®-coated mesoporous Pd film by simply dropping Nafion® perfluorinated resin solution onto a mesoporous Pd film. The uniform Nafion® coating can prevent Pd from redox reaction in NaCl solution. Furthermore, by applying an appropriate external potential, serious interferents present in human sweat, such as glucose, urea, ammonia and ethanol, can be minimized so the lactic acid concentration can be determined precisely. Such non-invasive electrochemical sensing based on Nafion®-coated mesoporous metal film is promising for daily diagnosis of various diseases and health management.

## Conflicts of interest

There are no conflicts to declare.

## Supplementary Material

RA-008-C7RA13026K-s001
