# Supplementary material for: Nafion®-coated mesoporous Pd film toward remarkably enhanced detection of lactic acid
Source: RSC Adv. 2018 Mar 14;8(19):10446–9. doi: 10.1039/c7ra13026k (PMC9078978; doi:10.1039/c7ra13026k)
Supplement: RA-008-C7RA13026K-s001 [file RA-008-C7RA13026K-s001.pdf]

Electronic Supplementary Material (ESI) for RSC Advances.
This journal is © The Royal Society of Chemistry 2018

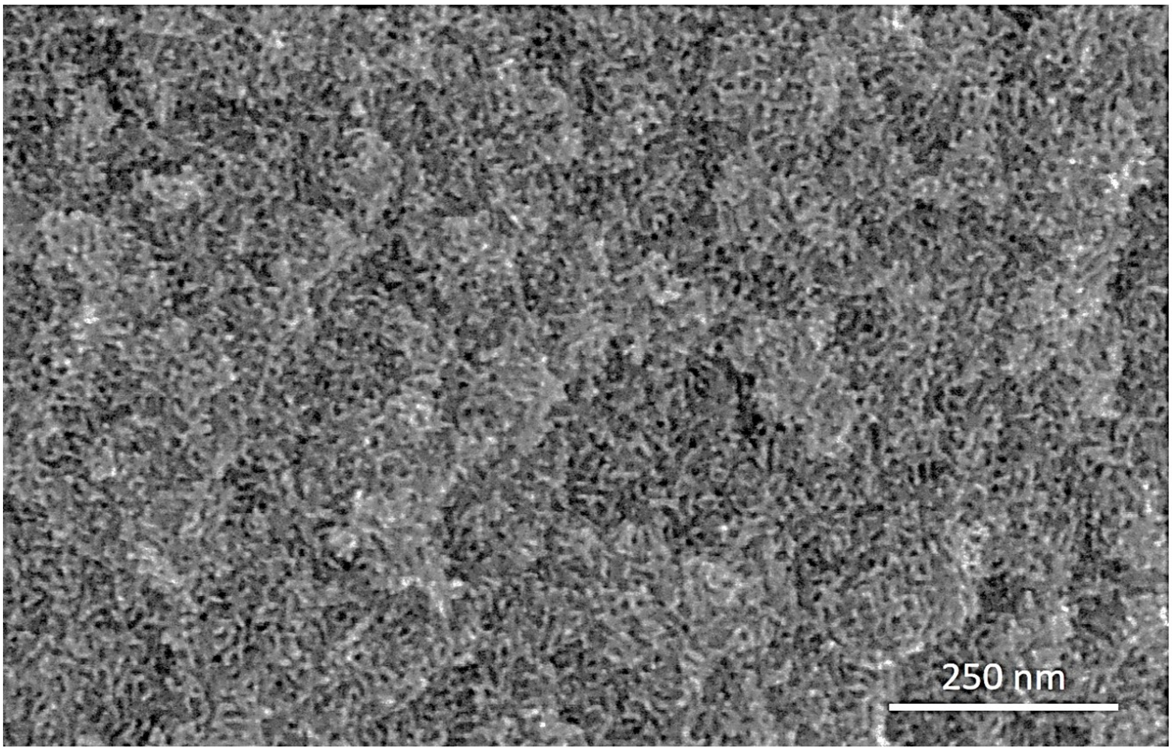

Figure S1 High resolution cross-sectional SEM image of mesoporous Pd film.

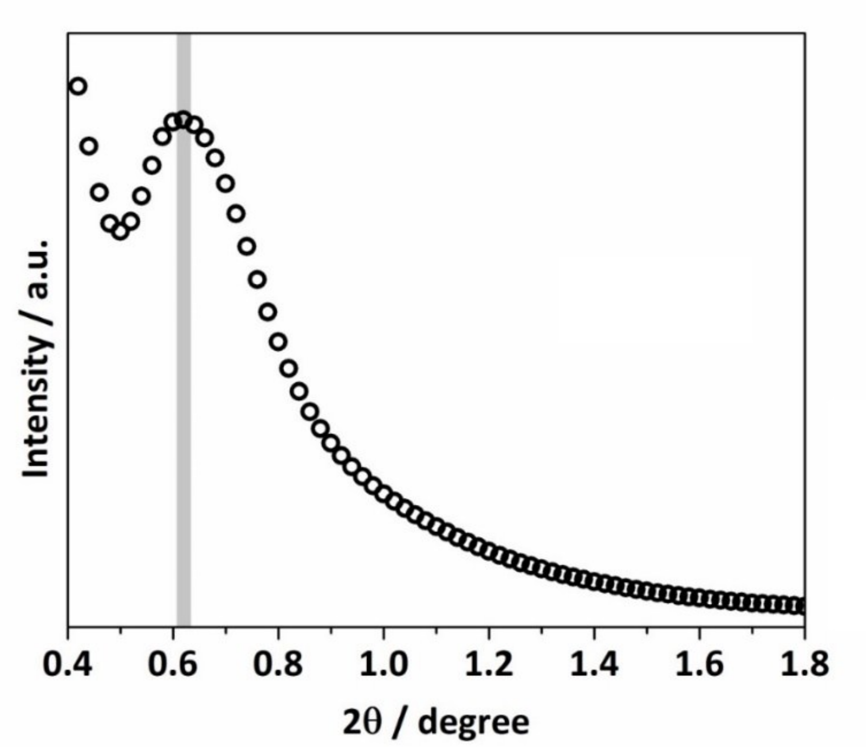

Figure S2 Low-angle XRD of mesoporous Pd film

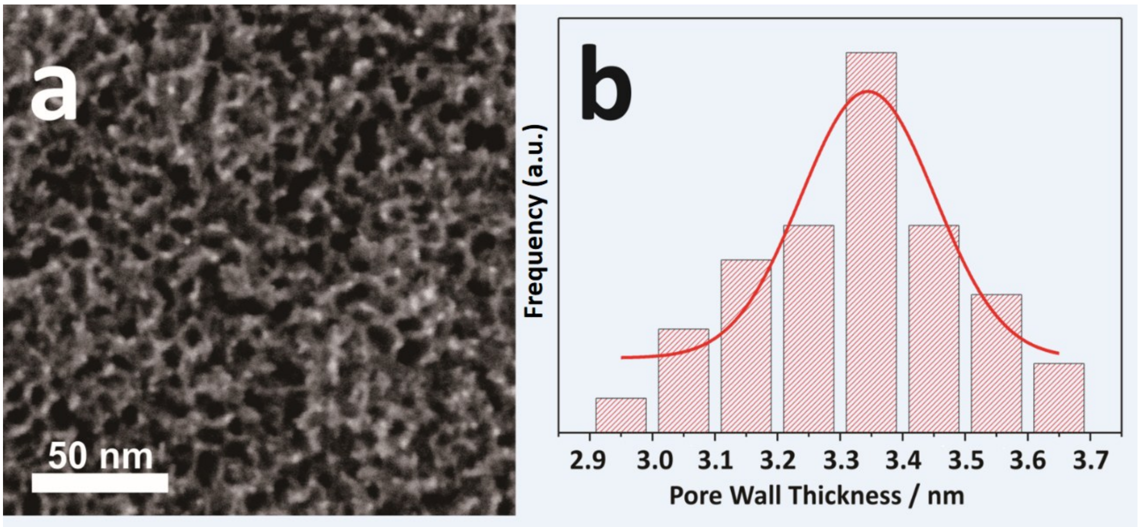

Figure S3 (a) High resolution SEM image of mesoporous Pd film. (b) Size distribution of pore wall thickness of mesoporous Pd film.

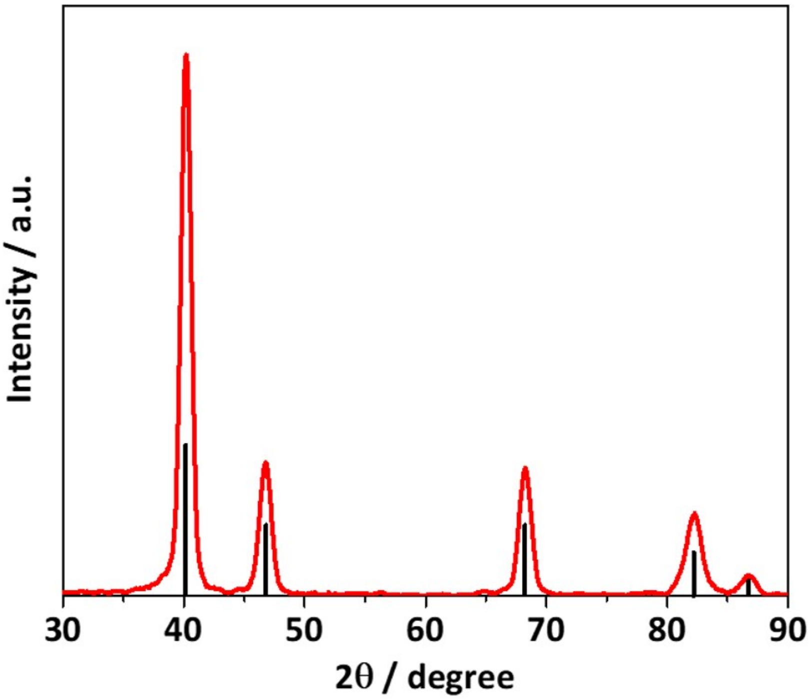

Figure S4 Comparison between the XRD pattern of the mesoporous Pd film and the JCPDS #05-0681.

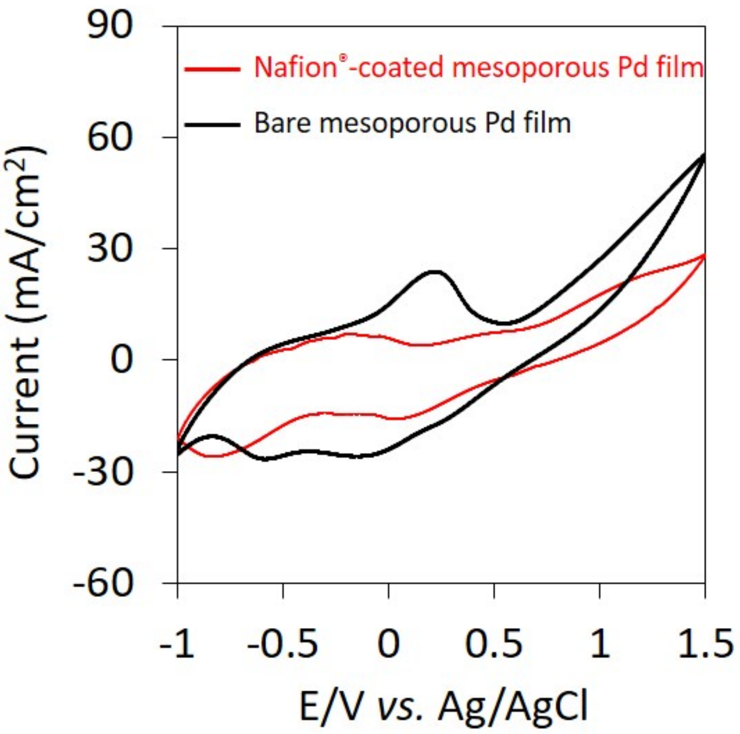

Figure S5 CV curve for Nafion®-coated mesoporous Pd film and bare mesoporous Pd film measured in NaCl solution (0.4 %, pH=7). The currents are normalized by geometric electrode area.

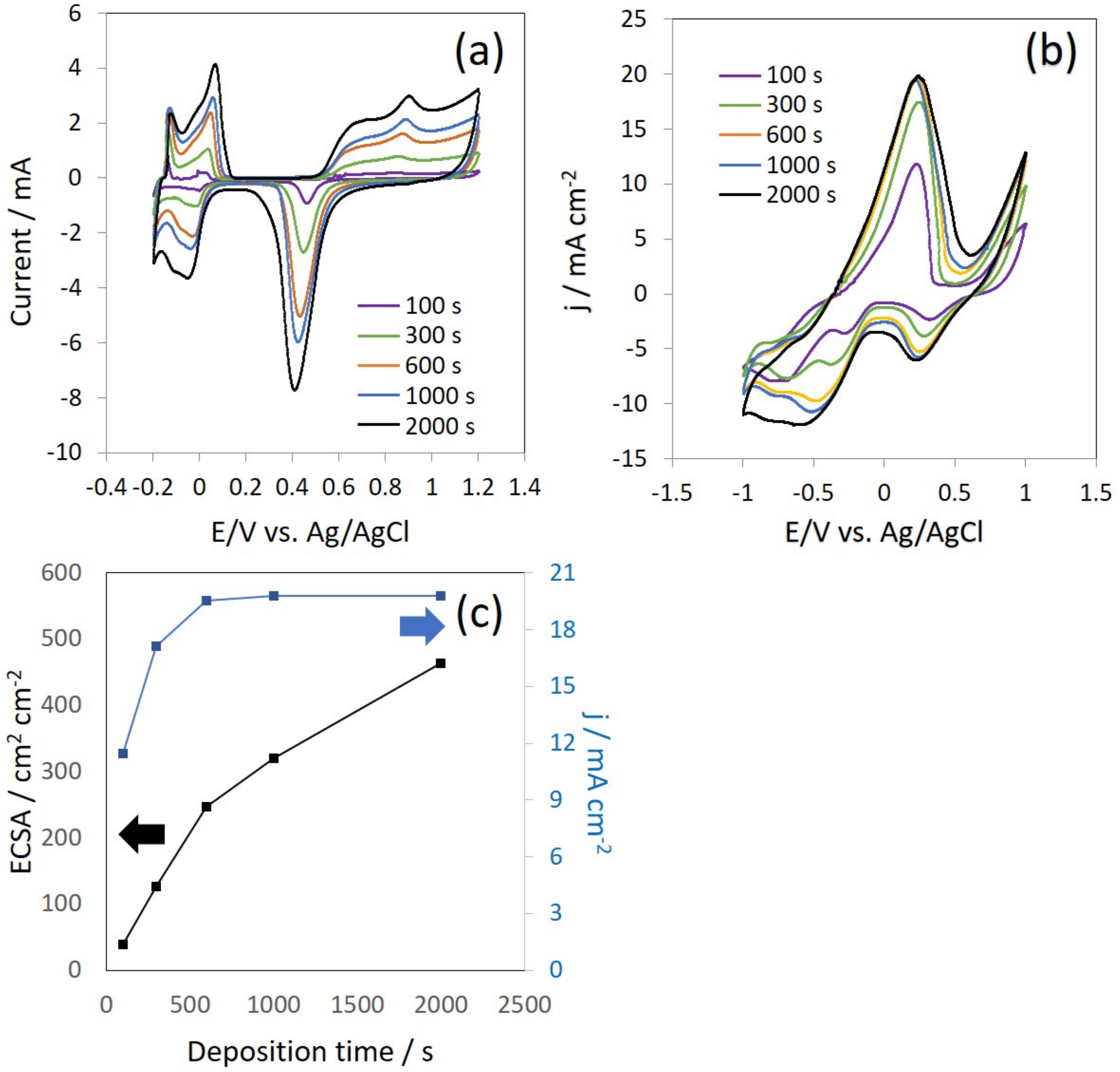

Figure S6 (a) CV curves of the mesoporous Pd films prepared at various deposition times (*i.e.*, with various thicknesses) in 0.5 M H_2SO_4. (b) CV curves measured in 0.4% NaCl solution containing lactic acid (10 mM). (c) Relationship between the deposition times *vs.* the ECSAs and the deposition times *vs.* the anodic peak current densities by oxidation of lactic acid. All the CV curves were obtained at a scan rate of 20 mV s^{-1}. The currents are normalized by geometric electrode area.

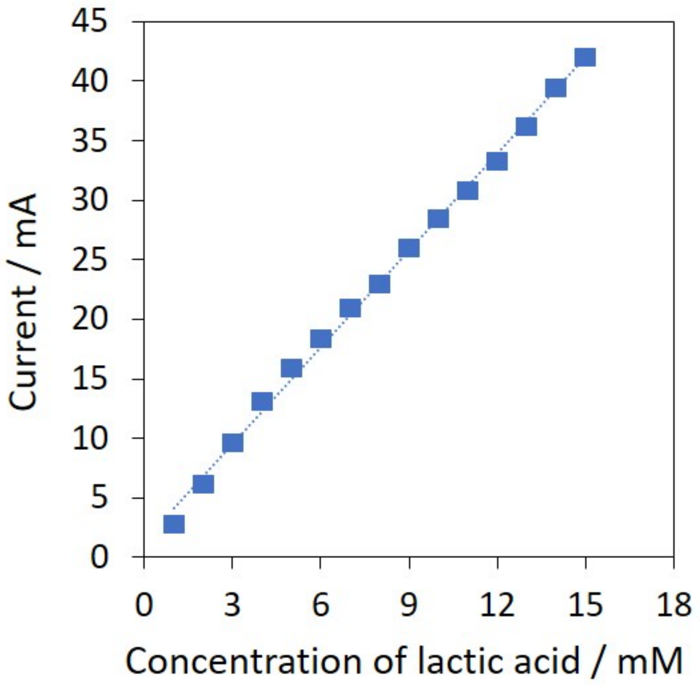

Figure S7 Relationship between the amperometric response and lactic acid concentrations (up to 15 mM in total, *i.e.*, 15 times drops).
